# Causal associations between primary sclerosing cholangitis and systemic lupus erythematosus: Evidence from Mendelian randomization and transcriptomic analyses

**DOI:** 10.1097/MD.0000000000045525

**Published:** 2025-10-31

**Authors:** Ying Wang, Zhe Zhou, Lei Jin, Hai-Ping Zhang

**Affiliations:** a Department of Nephrology & Rheumatology, Hubei NO.3 People’s Hospital of Jianghan University, Wuhan city, Hubei Province, China; b Department of Radiology, The Affiliated Hospital of Wuhan Sports University, Wuhan city, Hubei Province, China; c Department of Gastroenterology, Hubei NO.3 People’s Hospital of Jianghan University, Wuhan city, Hubei Province, China.

**Keywords:** autoimmune diseases, Mendelian randomization, primary sclerosing cholangitis, systemic lupus erythematosus, transcriptomic analysis

## Abstract

Overlap between primary sclerosing cholangitis (PSC) and systemic lupus erythematosus (SLE) has been documented in previous studies, but the precise causal relationships between them remain elusive. This study aims to clarify the potential causal links between PSC and SLE using Mendelian randomization (MR) and transcriptomic analyses. Genome-wide association study (GWAS) summary data for PSC and SLE were obtained from the IEU OpenGWAS database. The inverse variance weighted (IVW) method was the primary approach for assessing the causal links between PSC and SLE. Potential horizontal pleiotropy and heterogeneity were evaluated to ensure the reliability of the MR results. Additionally, transcriptomic analysis was performed using data from the Gene Expression Omnibus (GEO) database to uncover potential mechanisms insights involving overlapping genes and develop a diagnostic model for PSC and SLE. Genetically predicted PSC had a significant causal effect on SLE, with an OR of 1.190 (95% CI: 1.098–1.290, *P* ˂.001). Conversely, SLE had causal effect on PSC with an OR of 1.130 (95% CI: 1.046–1.221, *P* = .002). Multivariate MR analysis by adjustment of body mass index and smoking also revealed the bidirectional causal relationships between PSC and SLE. There were no signs of horizontal pleiotropy or heterogeneity, and the robustness of these results was confirmed via leave-one-out sensitivity analysis. Through integrated transcriptomic analysis and machine learning algorithms, 5 hub genes were identified. Furthermore, the diagnostic accuracy of the 5-gene signature was confirmed via Nomogram, calibration curve, and decision curve analysis, highlighting the diagnostic potential of these hub genes for both PSC and SLE. In conclusion, our MR study successfully confirms the bidirectional causal relationships between PSC and SLE, shedding light on the intertwined nature of these 2 conditions. Moreover, we identified 5 hub genes that potentially mediate the overlap between PSC and SLE, providing valuable insights for early diagnosis and future mechanistic exploration.

## 1. Introduction

Systemic lupus erythematosus (SLE) is a complex autoimmune disease, which has the potential to cause severe damage to multiple organs.^[1]^ The incidence of SLE has increased over the past few decades, ranging from 0.3 to 31.5 cases per 100,000 people per year.^[2]^ SLE is frequently associated with other immune diseases such as Sjögren syndrome, autoimmune thyroid disease and inflammatory bowel disease.^[3,4]^ Although the liver is not a primary target organ for SLE, a substantial proportion of SLE patients display irregular liver function.^[5]^ Liver involvement in SLE has received considerable scientific attention.^[6,7]^ Importantly, the diagnosis of hepatic involvement in SLE may be delayed, which can impede the implementation of timely intervention to prevent the progression of hepatic involvement. A recent study showed that SLE patients with autoimmune liver diseases exhibit a unique pattern of clinical features, characterized by late-onset age, lung involvement, high immunoglobulin levels and abnormal liver function.^[8]^ Despite recent advancements in diagnostic techniques, there are still challenges in accurately distinguishing primary liver diseases from SLE.

Primary sclerosing cholangitis (PSC) is a progressive liver disease characterized by inflammation, fibrosis, and stricture of the intrahepatic and extrahepatic bile ducts, which may ultimately result in liver cirrhosis and liver failure. PSC is male-predominant, and the prevalence of PSC is increasing in Europe.^[9,10]^ The estimated prevalence rate of PSC is between 0.78 and 31.7 cases per 100,000 individuals.^[10]^ In addition, PSC is frequently accompanied by extrahepatic autoimmune diseases,^[11]^ with 75% of patients with PSC exhibiting concomitant inflammatory bowel disease.^[12]^ Currently, efficacious medical treatment for PSC is lacking, and liver transplantation remains the only life-extending treatment option for PSC patients. Given the paucity of therapeutic options, effective monitoring and early diagnosis of PSC are greatly needed.

Although the co-occurrence of SLE and PSC is considered extremely rare, several case reports have been published.^[13–17]^ Additionally, a Swedish PSC cohort reported a 1.7% incidence of SLE.^[18]^ However, the causal relationship between PSC and SLE remains unknown, necessitating further investigation to determine causality and develop diagnostic strategies. Whether this overlap suggests shared pathogenic pathways between the 2 diseases is yet to be clarified.

It is challenging to draw valid conclusions about the causal associations between PSC and SLE in traditional observational studies due to unadjusted confounders and reverse causality. Based on large sample genome-wide association study (GWAS) data, Mendelian randomization (MR) has been proven to be an effective technique in epidemiological research for evaluating the causal effects of exposure on outcomes.^[19]^ Unlike traditional observational studies, MR is less vulnerable to confounders because the allocation of genetic variants occurs at conception. Besides, transcriptomic analysis of overlapping diseases is useful for identifying hub genes that are involved in the pathogenesis of overlapping diseases. For instance, a recent MR study assessed the association between SLE and primary biliary cholangitis (PBC), revealing a bidirectional causal association between SLE and PBC.^[20]^ Further transcriptomic analysis identified overlapping hub genes of SLE and PBC,^[20]^ offering insights for future investigations into the common mechanisms between these diseases.

In the present study, we aimed to explore the causal association between PSC and SLE using MR analysis, which revealed bidirectional causal associations between PSC and SLE. Subsequently, the overlapping genes between PSC and SLE were identified through transcriptomic analysis. Machine learning algorithms were then applied to develop a diagnostic model for early detection and clinical intervention.

## 2. Materials and methods

### 2.1. GWAS summary data

The GWAS summary data for PSC patients (containing 2871 cases and 12019 controls)^[21]^ and SLE patients (5201 cases and 9066 controls)^[22]^ were acquired from the IEU OpenGWAS project (https://gwas.mrcieu.ac.uk/). PSC was diagnosed according to the established criteria, which encompass a comprehensive range of clinical, biochemical, radiological and histological assessments.^[23]^ All SLE cases were documented in accordance with the American College of Rheumatology classification criteria. Additionally, multivariate MR (MVMR) analysis was conducted to control for potential confounders such as body mass index (BMI)^[24]^ and smoking.^[25]^ All the included data are widely available to researchers, ethical approval and informed consent were obtained in each original publication. Hence, no additional ethical approval was needed.

**Figure 1.**
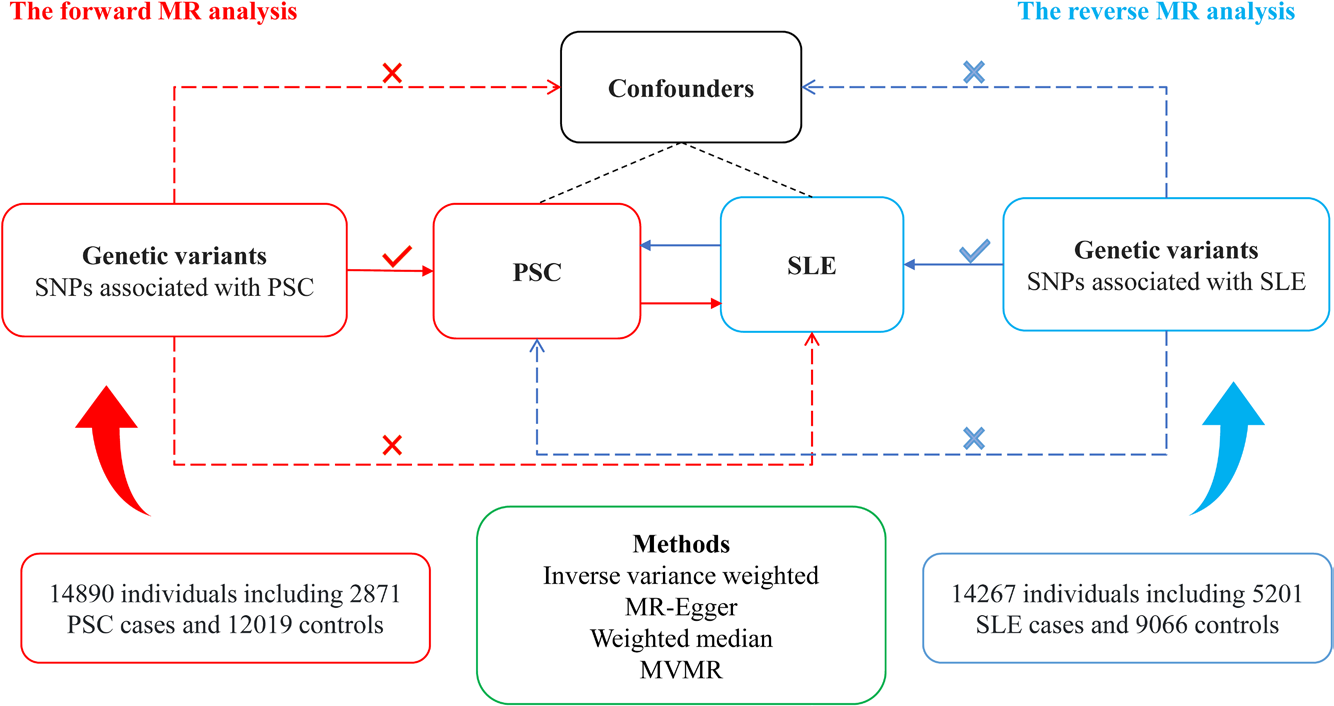
Study design for estimating associations between PSC and SLE. MVMR = multivariate Mendelian randomization, PSC = primary sclerosing cholangitis, SLE = systemic lupus erythematosus, SNP = single-nucleotide polymorphisms.

Our research adhered to the foundational assumptions of MR studies: the instrumental variables (IVs) showed a robust association with the exposure; the IVs had no connections with any potential confounders; and the influence of the IVs on the outcome was mediated solely through their relationship with the exposure (Fig. 1). Consequently, the selected single-nucleotide polymorphisms (SNPs) were significant at the genome-wide level (*P* < 5 × 10^−8^) and exhibited no linkage disequilibrium (*r*^2^ < 0.001 and kb < 10,000). To mitigate the influence of weak IVs, SNPs exhibiting an *F*-statistic lower than 10 were excluded.^[26]^ The LDlink database^[27]^ was used to exclude SNPs that are related to confounders, removing potential confounding effects on casual estimates.

### 2.2. MR analysis

The inverse variance weighted (IVW) approach was performed as the primary analysis using the TwoSampleMR package in R software (version 4.3.1). IVW combines the individual causal effects from each SNP to obtain a pooled estimate, assuming that all the SNPs are valid IVs. MR-Egger is performed to detect and adjust for pleiotropic effects where genetic variants influence the outcome through pathways other than the exposure. The weighted median approach provides a robust estimate when more than 50% of the weight is derived from valid SNPs. The outlier test was performed using the MR-PRESSO package, and subsequent causal estimates were calculated after outlier removal. The MR–Egger intercept test and global test of MR-PRESSO were used to assess the possible pleiotropic effects of the SNPs, and heterogeneity was assessed by Cochran *Q* test. Fixed effect IVW was applied if the *P* value of the Cochran *Q* test was < 0.05, otherwise random effect IVW was performed. In addition, leave-one-out analysis was implemented to enhance the reliability of the causal inference derived from the MR analysis. The “mv_multiple” function of TwoSampleMR package was applied to perform MVMR analysis to exclude the potential pleiotropic effects of confounder.

### 2.3. Identification of overlapping genes and functional analyses

The gene expression profiles and clinical data of GSE177044 (containing 43 PSC samples and 77 healthy controls) and GSE49454 (containing 157 SLE samples and 20 healthy controls) were obtained from the Gene Expression Omnibus (GEO) database. Differentially expressed genes (DEGs) of SLE and PSC were identified by the Limma package, with an absolute log2-fold change (|log2 FC|) >0.5 and adjusted *P* value* *< .05. Gene ontology (GO) enrichment analyses were performed to explore biological function, and an adjusted *P* value <.05 was considered indicative of significant enrichment. Additionally, gene set enrichment analysis (GSEA) was also performed to explore the biological pathways by the clusterProfiler package, with a threshold of adjusted *P* value* *< .05.

### 2.4. Nomogram establishment for PSC and SLE

After DEGs screening of both PSC and SLE, overlapping DEGs were subjected to least absolute shrinkage and selection operator (LASSO) with 10-fold cross validation to identify common hub DEGs. Receiver operating characteristic (ROC) curves were plotted to assess the diagnostic value of these hub genes. Risk scores were calculated using multivariate logistic regression with the shared hub genes. The risk scores for individual samples were determined based the expressions of common hub DEGs and corresponding logistic regression coefficients. The rms package was used to create a nomogram to improve the usability and practicality of the risk models. Calibration curves were plotted to estimate the model performance, providing insights into potential overfitting or underfitting. Decision curves were also plotted to assess the clinical utility by integrating the costs and benefits of different decision thresholds, helping to determine the value of the model in real-world decision-making scenarios.

## 3. Results

### 3.1. Causal effect of PSC on SLE

**Figure 2.**
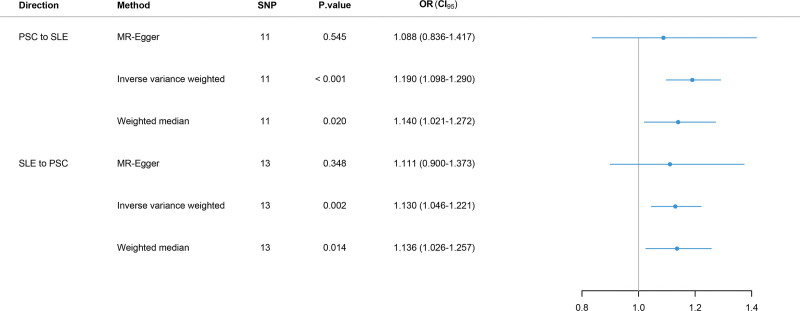
Forest plot of the bidirectional MR results. MR = Mendelian randomization.

**Figure 3.**
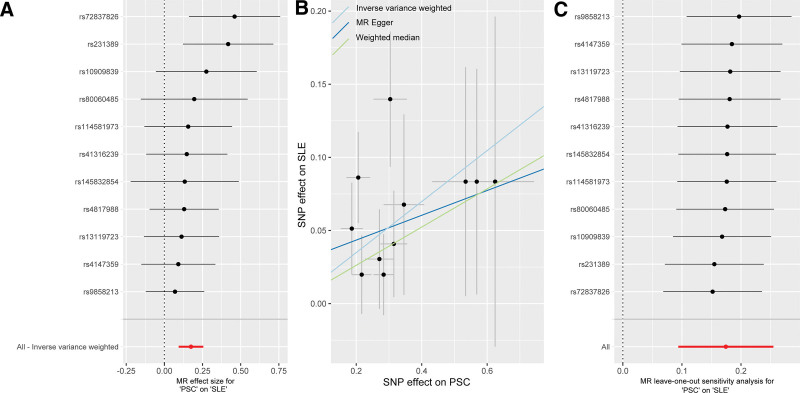
Causal effect of PSC on SLE. (A) Forest plot; (B) Scatter plot; (C) leave-one-out test. PSC = primary sclerosing cholangitis, SLE = systemic lupus erythematosus.

There were no overlapping individuals between 2 GWAS datasets. After conducting strict quality control measures, 11 SNPs were included in the MR analysis (Table S1, Supplemental Digital Content, https://links.lww.com/MD/Q501). The SNPs significantly associated with PSC at the genome-wide significance threshold (*P* < 5 × 10^−8^) were selected as IVs. Their *F*-statistics ranged from 30.1 to 83.7, substantially exceeding the standard threshold of *F* > 10, thereby minimizing weak instrument bias. Genetically predicted PSC was found to have a causal effect on SLE according to the IVW analysis (OR = 1.190, 95% CI: 1.098–1.290, *P* ˂ .001) (Figs. 2 and 3A). The weighted median approach also showed a positive association between PSC and SLE (OR = 1.140, 95% CI: 1.021–1.272, *P* = .02). Additionally, MR-Egger analysis revealed a same direction of causal effect on SLE (OR = 1.088, 95% CI: 0.836–1.417, *P* = .54) (Fig. 3B). The included SNPs did not exhibit any heterogeneity or horizontal pleiotropy in this MR study (Table S2, Supplemental Digital Content, https://links.lww.com/MD/Q501), suggesting adherence to MR’s core assumptions. These MR analysis results were also confirmed for robustness via the leave-one-out method (Fig. 3C).

### 3.2. Causal effect of SLE on PSC

**Figure 4.**
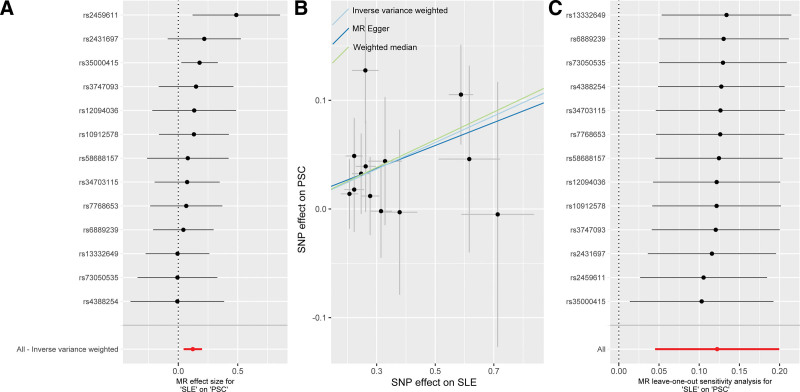
Causal effect of SLE on PSC. (A) Forest plot; (B) Scatter plot; (C) leave-one-out test. PSC = primary sclerosing cholangitis, SLE = systemic lupus erythematosus.

To estimate the causal effect of SLE on PSC, 13 SNPs associated with SLE were utilized (Table S3, Supplemental Digital Content, https://links.lww.com/MD/Q501). All SNPs were significantly associated with SLE with genome-wide significance *P* value < 5 × 10^−8^. Their *F*-statistics ranged from 32.2 to 200.2, indicating absence of weak instrument bias. The IVW method demonstrated that SLE had a causal effect on PSC (OR = 1.130, 95% CI: 1.046–1.221, *P* = .002) (Figs. 2 and 4A). Weighted median showed consistent result (OR = 1.136, 95% CI: 1.026–1.257, *P* = .01). Similarly, the MR-Egger (OR = 1.111, 95% CI: 0.900–1.373, *P* = .35) showed the same direction of causal effect (Fig. 4B). No heterogeneity or horizontal pleiotropy was observed among the included SNPs (Table S2, Supplemental Digital Content, https://links.lww.com/MD/Q501), which confirmed adherence to MR’s core assumptions. These MR analysis results were also confirmed for robustness via the leave-one-out method (Fig. 4C).

### 3.3. MVMR analysis

Previous studies have shown associations of these 2 diseases with BMI and smoking.^[28–30]^ After adjusting for BMI and smoking, significant causal association was found between PSC and an elevated risk of SLE, with an OR of 1.161 (95% CI: 1.059–1.273, *P* = .001). On the other hand, SLE increased the risk of PSC (OR = 1.578, 95% CI: 1.409–1.767, *P* < .001) following adjustments for BMI and smoking (Table S4, Supplemental Digital Content, https://links.lww.com/MD/Q501). The bidirectional associations indicate potential shared pathophysiological mechanisms or genetic predispositions between PSC and SLE, underscoring the importance of further investigations into the link between these 2 autoimmune conditions.

### 3.4. DEG identification and functional analysis

**Figure 5.**
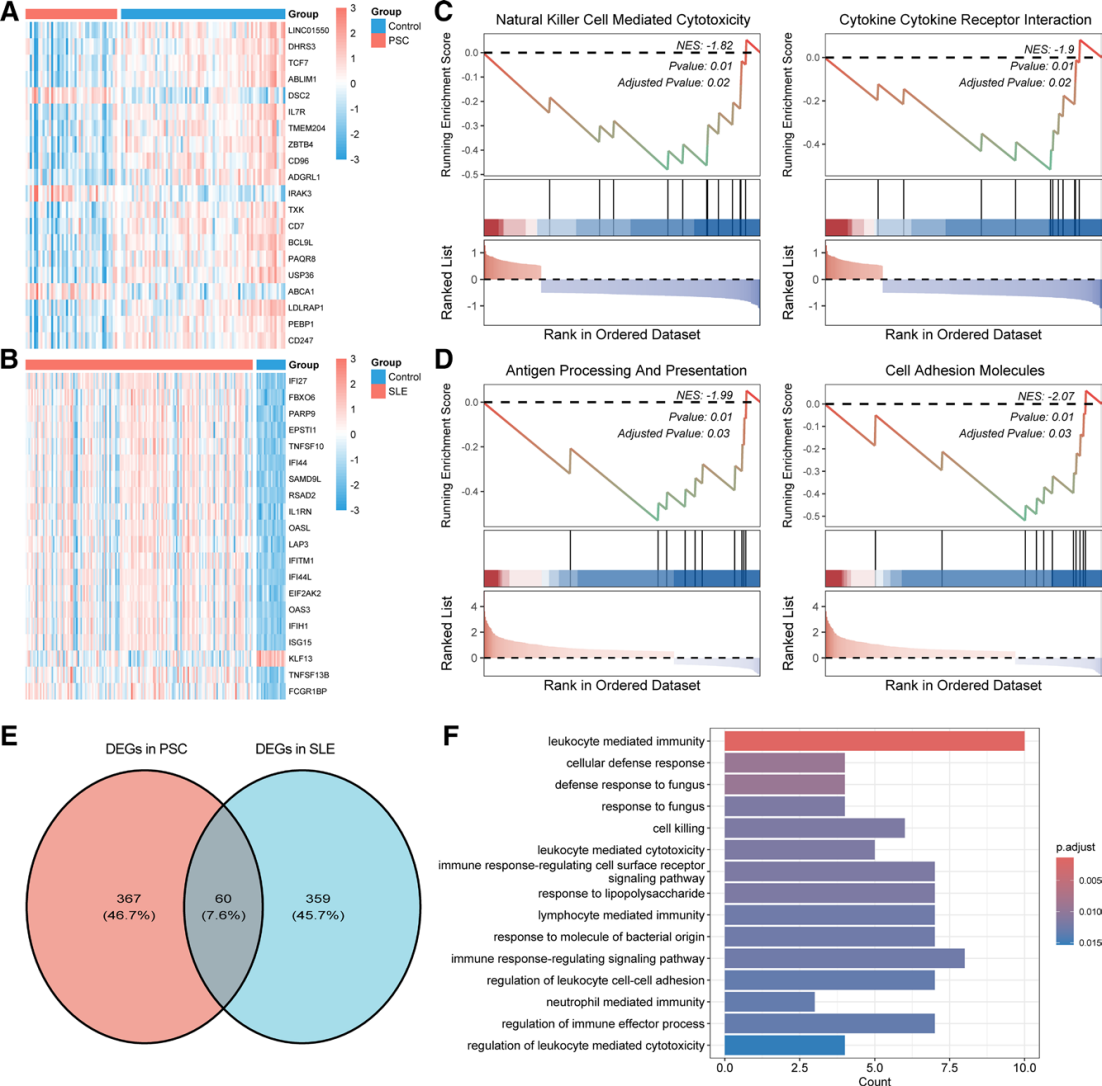
DEG identification and functional analysis. (A and B) Top 20 DEGs in the PSC and SLE datasets; (C and D) GSEA results of DEGs in PSC and SLE datasets; (E) Venn diagram of 60 overlapping DEGs; (F) GO enrichment results of 60 overlapping DEGs. DEG = Differentially expressed gene, GO = gene ontology, GSEA = gene set enrichment analysis, PSC = primary sclerosing cholangitis, SLE = systemic lupus erythematosus.

A total of 427 DEGs were identified in PSC (85 upregulated genes and 342 downregulated genes), and 419 DEGs were identified in SLE (288 upregulated genes and 131 downregulated genes) (Tables S5 and S6, Supplemental Digital Content, https://links.lww.com/MD/Q501). The top 20 DEGs were shown in Figure 5A and B. The GSEA results indicated that DEGs in PSC mainly participated in cytokine and cytokine receptor interaction, as well as natural kill cell mediated cytotoxicity (Fig. 5C). DEGs in SLE primarily participated in cell adhesion and molecules and antigen processing and presentation (Fig. 5D). There were 60 overlapping DEGs between SLE and PSC (Fig. 5E). GO enrichment analysis showed the significant role of the 60 genes in immune-related processes, including leukocyte mediated immunity, immune response-regulating signaling pathway, leukocyte mediated immunity, and regulation of leukocyte cell–cell adhesion (Fig. 5F, Table S7, Supplemental Digital Content, https://links.lww.com/MD/Q501). These findings reinforced the notion that dysregulated immune responses are central to the pathogenesis of these autoimmune diseases.

### 3.5. Hub DEGs in PSC and SLE

**Figure 6.**
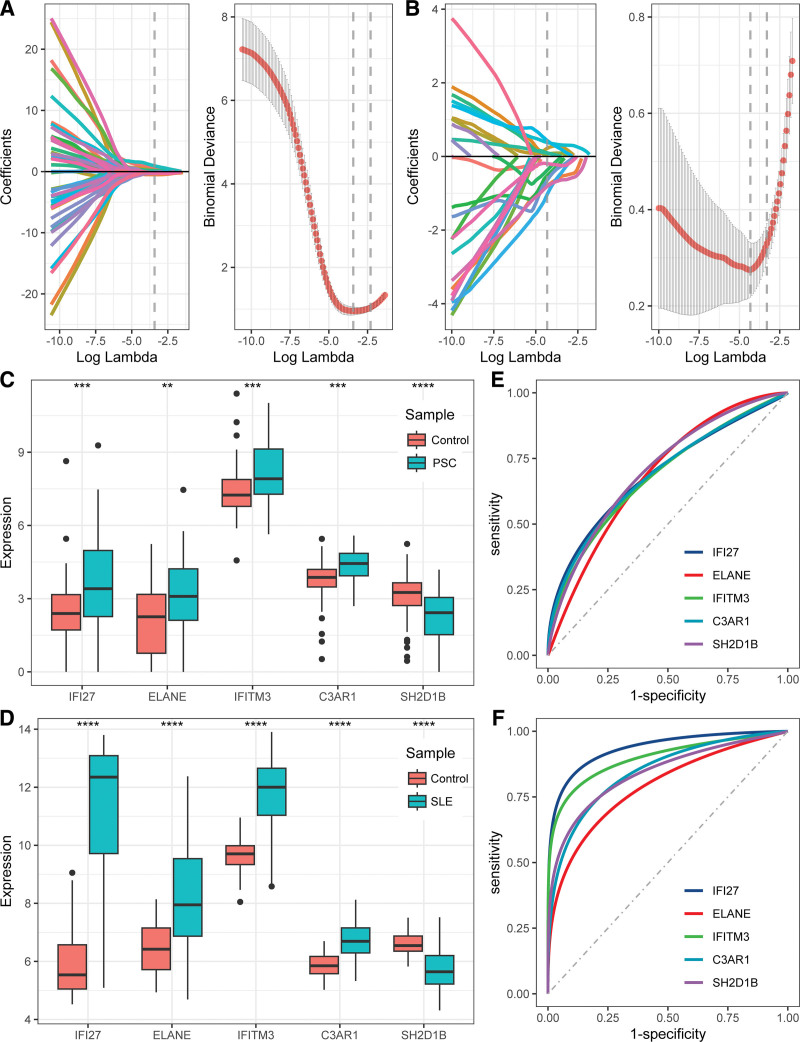
Hub gene selection. (A) LASSO analysis of the PSC dataset; (B) LASSO analysis of the SLE dataset; (C and D) expression levels of 5 genes in the PSC and SLE datasets; (E and F) ROC curves of 5 genes in the PSC and SLE datasets. LASSO = least absolute shrinkage and selection operator, PSC = primary sclerosing cholangitis, ROC = receiver operating characteristic, SLE = systemic lupus erythematosus.

In GSE49454, the LASSO regression algorithm identified 14 DEGs with an optimal λ value of 0.013 (Fig. 6A). In GSE177044, the LASSO method identified 17 DEGs with an optimal λ value of 0.033 (Fig. 6B). Five genes (IFI27, ELANE, IFITM3, C3AR1, and SH2D1B) were identified as hub DEGs for PSC and SLE. The expression levels of the 5 genes in the PSC and SLE datasets are shown in Figure 6C and D. IFI27, ELANE, IFITM3, and C3AR1 showed increased expression in both the PSC and SLE samples compared with the control samples, whereas SH2D1B exhibited decreased expression. Univariate logistic regression analysis showed that IFI27, ELANE, IFITM3, and C3AR1 were risk factors while SH2D1B was a protective factor for PSC and SLE (Table S8, Supplemental Digital Content, https://links.lww.com/MD/Q501). ROC curves showed good performance of the 5 genes in the PSC and SLE datasets (Fig. 6E and F).

### 3.6. Nomogram establishment for PSC and SLE

**Figure 7.**
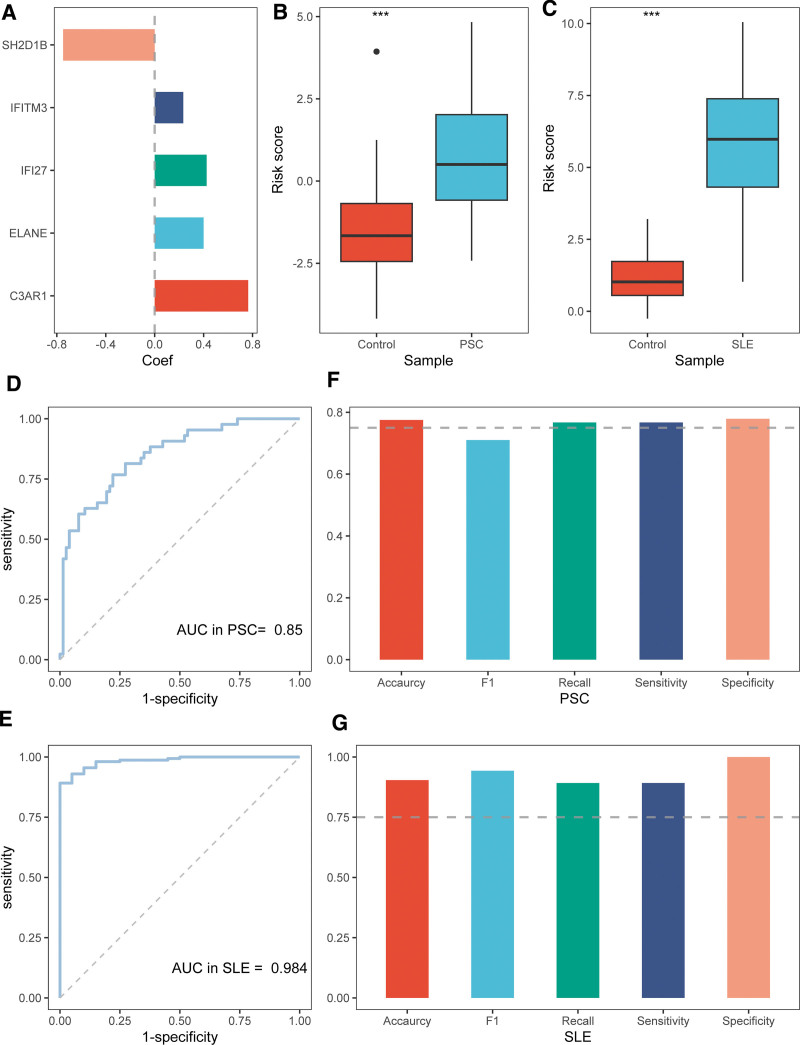
Risk scores in the PSC and SLE datasets. (A) Coefficients of 5 genes; (B and C) risk scores between cases and controls; (D and E) ROC curves of risk scores; (F and G) diagnostic performance of risk scores. PSC = primary sclerosing cholangitis, ROC = receiver operating characteristic, SLE = systemic lupus erythematosus.

The 5 hub DEGs were subsequently subjected to multivariate logistic regression to develop a diagnostic model for patients PSC and SLE patients. The risk scores were calculated by the expressions of 5 hub DEGs and the corresponding regression coefficients (Table S9, Supplemental Digital Content, https://links.lww.com/MD/Q501), Fig. 7A). The risk scores were higher in the PSC (SLE) samples than in the healthy control samples (Fig. 7B and C). The area under the curve values of the risk scores for diagnosing PSC and SLE were 0.85 and 0.984, respectively (Fig. 7D and E). The accuracy, sensitivity, and specificity of the 2 risk scores exceeded 0.75 (Fig. 7F and G). The risk score in SLE patients was associated with renal involvement and SLE disease activity index, indicating its potential for prognostic assessment (Fig. S1, Supplemental Digital Content, https://links.lww.com/MD/Q502). In addition, the score was related to various immune cells in patients with SLE (Fig. S2, Supplemental Digital Content, https://links.lww.com/MD/Q502), supporting the significance of the hub genes in immune regulation.

**Figure 8.**
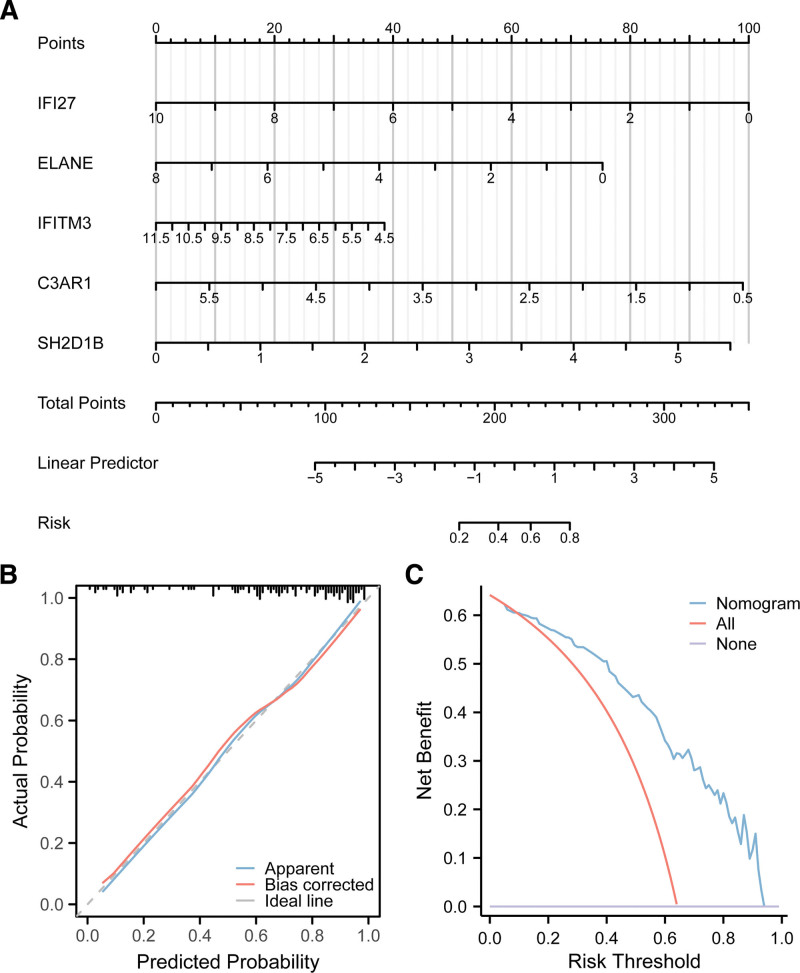
Nomogram establishment and evaluation. (A) Nomogram establishment in the PSC dataset; (B and C) calibration curves and decision curves.

A nomogram based on risk scores was developed to provide clinicians with a quantifiable method to predict the likelihood of PSC and SLE (Fig. 8A; Fig. S3A, Supplemental Digital Content, https://links.lww.com/MD/Q502). In both the PSC and SLE cohorts, the calibration curves indicated good consistency between the predicted and actual results (Fig. 8B; Fig. S3B, Supplemental Digital Content, https://links.lww.com/MD/Q502). With a wide range of threshold probabilities, the net benefit of the model was significantly greater than that of the “treat all” and “no treatment” baselines (Fig. 8C; Fig. S3C, Supplemental Digital Content, https://links.lww.com/MD/Q502). This finding indicated that within this threshold range, the model can more effectively guide clinical decision-making, reducing unnecessary interventions and misdiagnoses.

## 4. Discussion

Our research aimed to evaluate the potential link between PSC and SLE, which has not been addressed in previous studies. While clinically reported cases of overlap between PSC and SLE are rare, further research is necessary to determine whether their co-existence is coincidental or causal. Our study provides compelling evidence for the bidirectional causal associations between PSC and SLE. The current MR study did not exhibit heterogeneity or horizontal pleiotropy. The reliability of our MR findings was demonstrated by sensitivity analysis. Transcriptomic analysis revealed common genes in both PSC and SLE, suggesting potential overlap in the pathophysiological mechanisms of these autoimmune disorders. The risk model based on the 5 hub genes showed excellent power for diagnosing PSC and SLE. This research not only offers new insight into the accurate diagnosis of overlapping cases of PSC and SLE, but also lays the groundwork for future studies on shared mechanisms and potential treatment options.

Approximately 33% of individuals with autoimmune liver disease experience concomitant autoimmune conditions, which can affect the rheumatological, endocrinological, and gastrointestinal systems.^[31]^ Patients with PSC had a significantly greater rate of positive serum antibodies than controls, and 97% of PSC patients were positive for at least 1 antibody.^[32]^ Antinuclear antibodies are detected in 20% of patients with PSC.^[7]^ Although impaired liver function is often observed in SLE patients, there are few case reports on the co-occurrence of SLE and PSC. The co-existence of these 2 diseases can lead to more complicated organ involvement and poorer clinical prognosis.^[8]^ Using the MR approach, our research explored the causal links between PSC and SLE, revealing bidirectional relationships between the 2 conditions. By adjusting for BMI and smoking, the MVMR further validated the bidirectional causal association between PSC and SLE. Previous GWAS showed that PSC and SLE had overlapping genetic factors, such as SH2B3 and CLEC16A.^[21,22]^ Inflammatory cytokines play crucial roles in both diseases. Elevated levels of IL6, IL-17, and IFN-α/β are observed in patients with SLE,^[33]^ and increased IL6, IL-17 and INFγ levels are observed in PSC patients.^[34]^ The role of the gut microbiota in regulating the immune system is gaining attention. The composition of the gut microbiome in PSC and SLE patients may be altered, and these changes can promote the development of diseases by affecting both local and systemic immune responses.^[35,36]^ The above studies corroborate the results of our investigation, which indicated a potential genetic link between PSC and SLE.

Transcriptomic overlap analysis is useful for exploring common mechanisms between 2 diseases. For instance, the immune pathway of venous thromboembolism related to SLE has been revealed and a diagnostic model has been built, providing new insights for future serum diagnosis for SLE combined with venous thromboembolism.^[37]^ This study identified 60 overlapping genes that were found to be related to immune function via RNA sequencing data from SLE and PSC patients. Five genes (IFI27, ELANE, IFITM3, C3AR1, and SH2D1B) were identified as hub genes by further LASSO analysis. Among the 5 genes, SH2D1B has not been reported to be associated with SLE or PSC, and the other 4 genes (IFI27, ELANE, IFITM3, and C3AR1) have not been reported to be associated with PSC. IFI27 and IFITM3 were found to be associated with SLE through single-cell sequencing and RNA sequencing data analyses.^[38–40]^ ELANE and C3AR1 were reported to be related to renal involvement in SLE patients.^[41,42]^ Further research is needed to investigate the significance of shared hub genes to develop targeted therapies for PSC and SLE.

Given the findings from MR analysis, it is essential to implement effective screening strategies for early detection and management of these conditions. Patients diagnosed with either PSC or SLE should undergo regular monitoring for signs of the other condition, including liver function tests, antinuclear antibody tests, and imaging examinations. In the present study, we developed a diagnostic nomogram based on the risk scores of the above 5 hub genes, which effectively differentiated SLE and PSC patients from healthy controls. The calibration curves showed good consistency between our predictive values and the actual values, and decision curves showed great potential for application in clinical practice. While existing diagnostic criteria for SLE (e.g., ACR/EULAR criteria) and PSC (e.g., cholangiography) remain essential, our 5-gene risk model provides distinct clinical advantages as a complementary tool. First, it enables early detection by capturing molecular alterations prior to overt clinical manifestations, providing particularly valuable for seronegative or atypical presentations. Second, unlike conventional biomarkers (e.g., antinuclear antibody), our risk score correlates with renal involvement and SLE disease activity index in SLE, showing great potential for prognosis assessment. Finally, as a noninvasive blood-based tool, it could reduce dependency on costly/invasive procedures (e.g., ERCP for PSC) for initial screening or high-risk cohort monitoring. Future validation of these genes in serum or plasma may translate this model into a practical assay to stratify patients and guide targeted surveillance.

This study has several advantages. First, univariate MR and multivariate MR were performed to evaluate the bidirectional causal links between SLE and PSC, potentially reducing the influence of confounding factors. Second, this study employed transcriptome overlap analysis to confirm the potential shared mechanism between the 2. Finally, a diagnostic nomogram was established for clinical practice, indicating excellent performance for diagnosing SLE and PSC. Several shortcomings of the research warrant recognition in this study. First, the MR findings were drawn from the population of Europe, and it remains inconclusive whether there is a causal association between PSC and SLE in other ethnic groups. Second, individual information could not be obtained from GWAS summary data, and it was not possible to stratify the genetic-level causal effects between PSC and SLE by sex. Third, the transcriptomic data of the SLE and PSC datasets were obtained from different platforms. Finally, while our study supports bidirectional causality, we did not evaluate whether the included SNPs in the MR analysis directly regulate the expression of the shared hub genes. Future studies integrating eQTL data should elucidate these regulatory relationships to bridge genetic associations and transcriptional mechanisms.

## 5. Conclusion

The findings from the MR analysis indicate that there are bidirectional causal associations between PSC and SLE, which holds significant clinical guidance for regularly screening SLE in patients with PSC in daily medical practice and vice versa. Five genes (IFI27, ELANE, IFITM3, C3AR1, and SH2D1B) were identified as potential shared diagnostic biomarkers, offering valuable insights for investigating their underlying mechanisms. The diagnostic nomogram based on the 5 hub genes provided high accuracy in diagnosing PSC and SLE, which is helpful for the early diagnosis of these 2 diseases.

## Author contributions

**Conceptualization:** Ying Wang, Hai-Ping Zhang.

**Formal analysis:** Ying Wang, Zhe Zhou, Lei Jin, Hai-Ping Zhang.

**Methodology:** Ying Wang, Lei Jin, Hai-Ping Zhang.

**Software:** Ying Wang, Zhe Zhou, Lei Jin, Hai-Ping Zhang.

**Validation:** Ying Wang, Zhe Zhou, Lei Jin, Hai-Ping Zhang.

**Visualization:** Ying Wang, Zhe Zhou, Hai-Ping Zhang.

**Writing – original draft:** Ying Wang, Hai-Ping Zhang.

**Writing – review & editing:** Lei Jin, Hai-Ping Zhang.

## Supplementary Material




