# Supplementary material for: Causal associations between primary sclerosing cholangitis and systemic lupus erythematosus: Evidence from Mendelian randomization and transcriptomic analyses
Source: Medicine (Baltimore). 2025 Oct 31;104(44):e45525. doi: 10.1097/MD.0000000000045525 (PMC12582807; doi:10.1097/MD.0000000000045525)
Supplement: Supplementary file 2 [file medi-104-e45525-s002.pdf]

Supplementary Figure 1 Relationships between the risk score and clinical characteristics. Correlations of risk score with skin involvement (A), joints involvement (B), hematological involvement (C), renal involvement (D) and SLEDAI score (E, F) in SLE patients.

Supplementary Figure 2 Correlations with various immune cells in SLE patients.

Supplementary Figure 3 Nomogram establishment and evaluation in the SLE dataset. A, Nomogram establishment; B-C, Calibration curves and decision curves curves.

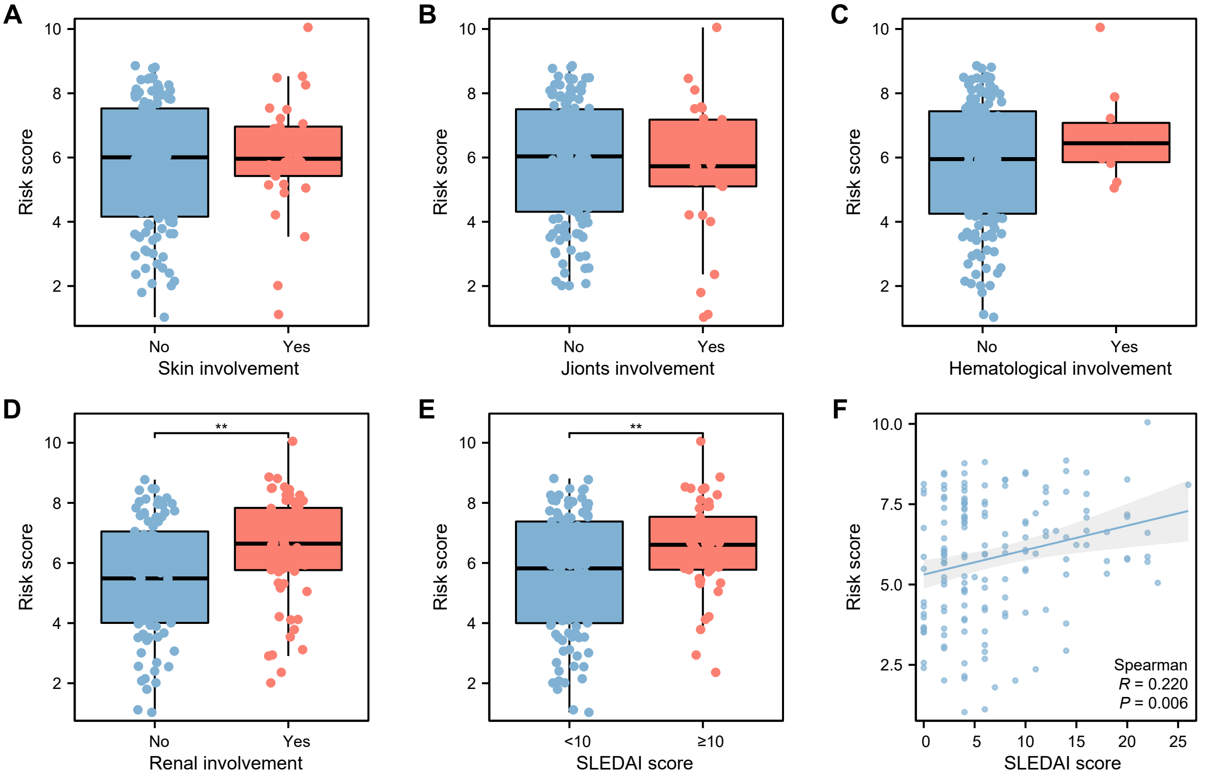
A
B
C
D
E
F
Risk score
No
Yes
Skin involvement
Jionts involvement
Hematological involvement
Renal involvement
**
<10
≥10
SLEDAI score
Spearman
R = 0.220
P = 0.006

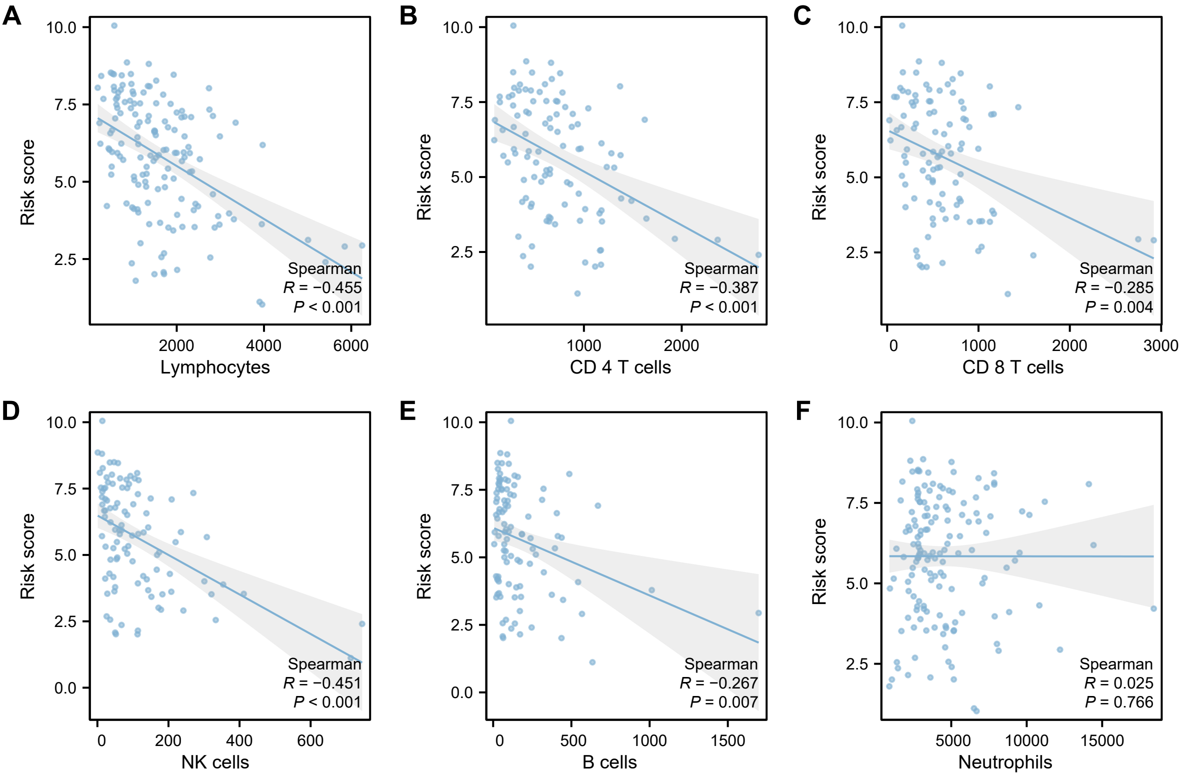

A
Risk score
Lymphocytes
Spearman
R = −0.455
P < 0.001
B
Risk score
CD 4 T cells
Spearman
R = −0.387
P < 0.001
C
Risk score
CD 8 T cells
Spearman
R = −0.285
P = 0.004
D
Risk score
NK cells
Spearman
R = −0.451
P < 0.001
E
Risk score
B cells
Spearman
R = −0.267
P = 0.007
F
Risk score
Neutrophils
Spearman
R = 0.025
P = 0.766

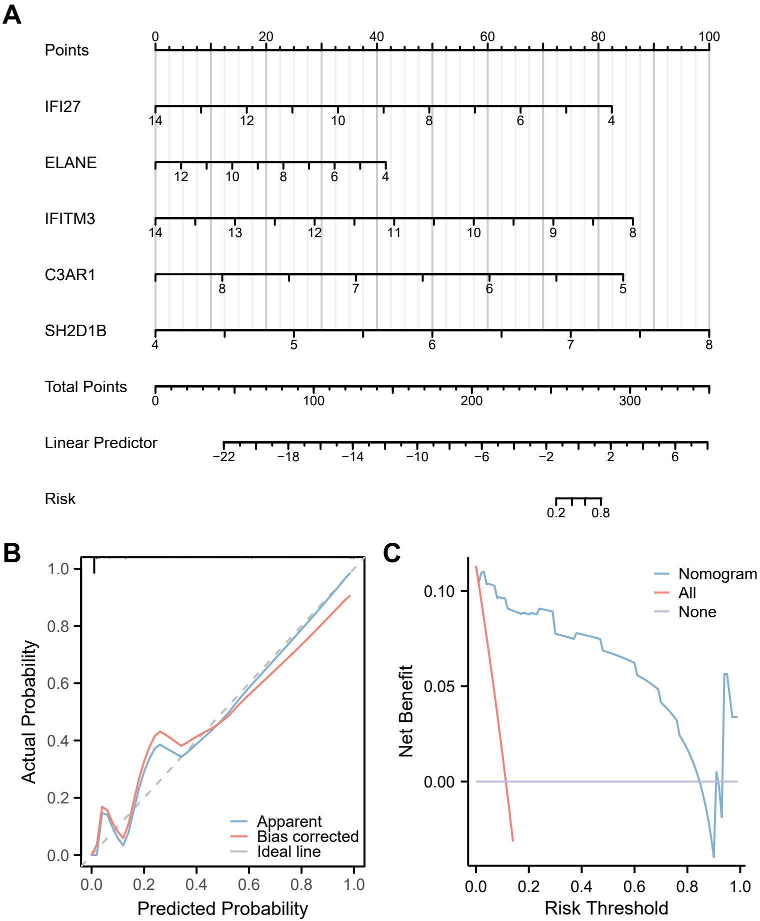
A
Points
IFI27
ELANE
IFITM3
C3AR1
SH2D1B
Total Points
Linear Predictor
Risk
B
Actual Probability
Predicted Probability
Apparent
Bias corrected
Ideal line
C
Net Benefit
Risk Threshold
Nomogram
All
None
